# Stereotactic Radiosurgery for Vestibular Schwannomas: Reducing Toxicity With 11 Gy as the Marginal Prescribed Dose

**DOI:** 10.3389/fonc.2020.598841

**Published:** 2020-10-29

**Authors:** Guillaume Dupic, Marie Urcissin, Thierry Mom, Pierre Verrelle, Véronique Dedieu, Ioana Molnar, Youssef El-Ouadih, Vincent Chassin, Michel Lapeyre, Jean-Jacques Lemaire, Julian Biau, Toufic Khalil

**Affiliations:** ^1^Department of Radiation Oncology, Jean Perrin Center, University of Clermont Auvergne, Clermont-Ferrand, France; ^2^Department of Otoneurolaryngology, Clermont-Ferrand Hospital, University of Clermont Auvergne, Clermont-Ferrand, France; ^3^Department of Medical Physics, Jean Perrin Center, University of Clermont Auvergne, Clermont-Ferrand, France; ^4^Department of Clinical Research, UMR 501, Jean Perrin Center, Clermont-Ferrand, France; ^5^INSERM U1240 IMoST, University of Clermont Auvergne, Clermont-Ferrand, France; ^6^Department of Neurosurgery, Clermont-Ferrand Hospital, University of Clermont Auvergne, Clermont-Ferrand, France

**Keywords:** stereotactic radiosurgery (SRS), vestibular schwannomas (VS), efficacy and safety, toxicity, dose de-escalation

## Abstract

**Background:**

Stereotactic radiosurgery (SRS) is a common treatment option for vestibular schwannomas. Historically, a dose de-escalation of the marginal prescribed dose from 16 Gy to 12–13 Gy has been done to limit toxicity without reducing local control (LC). We aimed to retrospectively report outcomes of Linac-based SRS for vestibular schwannomas treated with different doses.

**Methods:**

Included in the study were 97 stage 1 (1%), 2 (56%), 3 (21.5%), and 4 (21.5%) vestibular schwannomas treated with Linac-based (Novalis^®^) SRS from 1995 to 2019. No margin was added to the GTV to create the PTV. The median marginal prescribed dose was 14 Gy (range: 12–16 Gy) before 2006 and then 11 Gy for all patients (61 pts). Mean tumor volume was 1.96 cm^3^, i.e., about 1.6 cm in diameter. Mean follow-up was 8.2 years.

**Results:**

Following SRS, LC at 3, 5, and 10 years was 100%, 98.4%, and 95.6%, respectively [100% for those with ≤ 13 Gy as the marginal prescribed dose (NS)]. Toxicity to the trigeminal nerve was reported in 7.2% of cases (3.3% and 0% for transient and permanent toxicity for 11 Gy). The marginal prescribed dose was the only significant predictive factor in univariate and multivariate analysis (HR = 1.77, 95% CI = 1.07–3.10, p = 0.028). Toxicity to the facial nerve was reported in 6.2% of cases. The marginal prescribed dose was again the only significant predictive factor in univariate and multivariate analysis (HR = 1.31, 95% CI = 0.77–2.23, p = 0.049).

**Conclusion:**

Linac-based SRS for stages 1–3 vestibular schwannomas provides excellent outcomes: a 10-year LC rate of over 95%, with a permanent facial or trigeminal toxicity rate of under 5%. A marginal prescribed dose of 11 Gy seems to decrease nerve toxicity and facial toxicity in particular, without reducing LC. Prospective studies with longer follow-up are needed.

## Highlights

-Our study shows outcomes of Linac-based SRS for vestibular schwannomas with a mean follow-up of 8.2 years.
-11 Gy as marginal prescribed dose for vestibular schwannomas provides excellent local control (5-year LC = 100%).
-11 Gy as marginal prescribed dose for vestibular schwannomas seems to reduce cranial nerve toxicity.
-No permanent facial or trigeminal toxicity was observed for 11 Gy as marginal prescribed dose.
-Transient facial or trigeminal toxicity was observed in less than 5% for 11 Gy as marginal prescribed dose (0% and 3.2%, respectively).

## Introduction

Vestibular schwannomas are the most frequent tumors of cranial nerves with an incidence rate of 1 to 2 per 100,000 people. They are often unilateral, slightly more common in women and occur in patients with a mean age of 55 years (1, 2). In about 5% of cases, they are linked to genetic diseases [mainly neurofibromatosis type 2 (NF2)] and can be bilateral; such cases generally occur in patients aged around 30 years old (1). Vestibular schwannomas’ evolution is often slow and is classified according to the 4 stages of the Koos classification (3). The aim of treatment is local control (LC) while preserving the nerves of the pontocerebellar angle (especially the trigeminal and the facial nerves). For stage 1 (intra-canal) and 2 (extra-canal with invasion of the pontocerebellar angle without contact with the cerebellum or the brainstem) vestibular schwannomas which are progressive or symptomatic, either surgery or radiotherapy may be proposed without significant difference in terms of efficacy (LC ≈ 90%) (4).

Stereotactic radiosurgery (SRS) can be used to treat vestibular schwannomas with a large diameter of less than 3 cm (5–7). The first SRS were carried out in the 1990s with a mean prescribed marginal dose of 16 Gy (8–10). Historically, a de-escalation of the prescribed dose was then observed to reduce toxicities without reducing LC. Therefore, 12 to 13 Gy as the prescribed marginal dose in a single session is now generally accepted as a standard, allowing a 5-year LC rate of 95% and a facial and trigeminal nerve toxicity rate of 6%–8% (of which approximately 4% is transient toxicity) (11, 12). A dose-effect relationship is widely reported for nerve toxicity: the greater the dose is over 8 Gy, the greater the risk of toxicity (13). Significantly higher toxicity rates are reported in literature for mean received doses to the nerves of 12 Gy (14). Because of their anatomy and proximity to the tumor, doses received to the trigeminal and even more to the facial nerves are directly linked to the marginal prescribed dose despite new radiotherapy techniques for targeting the tumor and minimizing received doses to the surrounding tissue.

Therefore, our study aimed to retrospectively assess the efficacy and toxicity of Linac-based SRS for vestibular schwannomas with different marginal prescribed doses over time, to determine the influence of dose de-escalation and the best marginal prescribed dose in order to reduce toxicity while maintaining excellent LC.

## Patients and Methods

### Patients’ Selection and Characteristics

Ninety-seven patients (pts) consecutively treated for a vestibular schwannoma with Linac-based SRS from November 1995 to April 2019 were retrospectively included. Inclusion criteria were patients aged ≥ 18 years with performance status ≤ 2 and stages 1 to 4 vestibular schwannomas of the Koos classification with a large diameter of less than 3 cm. All patients were either symptomatic or with imaging proof of progression. Exclusion criteria were normo-fractionated stereotactic radiotherapy (FSRT) and schwannomas of another cranial nerve.

Initial damage to the facial nerve (determined using the House-Brackmann scale) and to the trigeminal nerve was evaluated as well as vestibular damage and damage to the brainstem (hydrocephalus in particular). Hearing function was assessed with the Gardner-Robertson scale: stages 1 and 2 corresponded to useful hearing and stages 3 to 5 to non-useful hearing. Oral corticosteroids (80 mg of prednisolone per day, gradually decreasing over 4 weeks) were delivered to patients the day of SRS.

All pre-treatment characteristics of the 97 included patients are reported in **Table 1**. The mean age was 64 years (range: 25–87 years). Patients were classified into 4 groups according to the marginal prescribed dose: 11, 12, 14, and 16 Gy, respectively. Initial injury to the trigeminal nerve was observed in 11 patients (11.3%): 3, 2, 4, and 2 for groups 11, 12, 14, and 16 Gy, respectively (NS). Damage to the facial nerve was also reported in 11 patients (11.3%): 4, 3, 3, and 1 groups 11, 12, 14, and 16 Gy, respectively (NS). Useful hearing was observed in 39 patients (41.1%) and a hydrocephalus in 6 patients (6.2%). Vestibular schwannomas were mostly stage ≤ 3 (78.4%). The mean GTV was 1.96 cc, i.e., about 1.6 cm in diameter (1.38 cc for groups 11–12 Gy and 3.52 cc for groups 14–16 Gy, p < 0.001).

**Table 1:** Patients, vestibular schwannomas, and SRS characteristics.

Marginal prescribed dose		AllN (%)	16 GyN (%)	14 GyN (%)	12 GyN (%)	11 GyN (%)
**Patients’ characteristics**						
Total		**97**	6	21	9	61
Gender						
	male	**40 (41%)**	3 (50%)	8 (38%)	6 (67%)	23 (38%)
	female	**57 (59%)**	3 (50%)	13 (62%)	3 (33%)	38 (62%)
Age (years)		**63.8**	57.1	61.3	70.2	64.4
Gene predisposition						
	NF2	**3 (3%)**	0 (0%)	1 (5%)	0 (0%)	2 (3%)
	no	**94 (97%)**	6 (100%)	20 (95%)	9 (100%)	59 (97%)
Trigeminal nerve deficit						
	yes	**11 (11%)**	2 (33%)	4 (19%)	2 (22%)	3 (5%)
	no	**86 (89%)**	4 (67%)	17 (81%)	7 (78%)	58 (95%)
Facial nerve deficit						
	yes	**11 (11%)**	1 (16%)	3 (14%)	3 (33%)	4 (7%)
	no	**86 (89%)**	5 (83%)	18 (86%)	6 (67%)	57 (93%)
Useful hearing						
	yes	**39 (41%)**	2 (33%)	5 (24%)	2 (22%)	30 (49%)
	no	**56 (59%)**	4 (67%)	16 (76%)	7 (78%)	31 (51%)
**Vestibular schwannomas’ characteristics**						
Total		**97**	6	21	9	61
GTV						
	mean (cc)	**1.96**	4.3	3.3	3.2	1.1
Stage						
	1	**1 (1%)**	0 (0%)	0 (0%)	0 (0%)	1 (1%)
	2	**54 (56%)**	2 (33%)	10 (48%)	2 (22%)	40 (66%)
	3	**21 (22%)**	2 (33%)	3 (14%)	3 (33%)	13 (21%)
	4	**21 (22%)**	2 (33%)	8 (38%)	4 (45%)	7 (12%)
Prior surgery						
	yes	**18 (19%)**	1 (17%)	6 (29%)	3 (33%)	8 (13%)
	no	**79 (81%)**	5 (83%)	15 (71%)	6 (67%)	53 (87%)
**Follow-up (months)**						
	mean	**98.7 (5.5**–**295.9)**	229.2 (25.8–295.9)	198.7 (14.0–256.3)	168.3 (106.1–215.6)	41.2 (5.5–158)

Bolded values are values of the entire population (all patients are concerned and not only a group like other columns).

### SBRT Specifications

From December 1995 to January 2011, SRS was performed using a Varian® Clinac 2100C (Varian Medical System, Palo Alto, CA) linear accelerator: with cylindrical collimators (diameter 6 to 24 mm) from 1995 to 2000 and with an additional micro multi-leaf collimator m3 Brainlab® (Brainlab, Feldkirchen, Germany) from 2000 to 2011. During this time, a Leksell stereotactic head frame was used (60 pts, 61.9%). From January 2011, SRS was performed with a Novalis Tx^®^ (Varian Medical Systems, Palo Alto, CA) linear accelerator with an integrated ExacTrac X-ray 6D system^®^ (BrainLAB AG, Feldkirchen, Germany) which enables pretreatment positioning. A frameless mask without invasive procedures was used (37 pts, 38.1%). Dose distributions were performed with 4-5 non-coplanar conformal arcs from July 1996 to January 2002 and with 4-5 non-coplanar dynamic arcs after January 2002. Brainlab® TPS were used: BrainScan® and IplanRT®, respectively, before and after January 2011. Volumetric modulated arc therapy (VMAT) with non-coplanar arcs (Eclipse^®^, Varian, VMAT Eclipse®) was used on a case-by-case basis after 2018.

The gross tumor volume (GTV) was identified using 0.9-mm 3D-CISS, T2-weighted and gadolinium-enhanced axial magnetic resonance imagery (MRI) sequences fused with high-resolution (1.25-mm slice thickness) computed tomography (CT) images. All 97 patients were treated with single-fraction SRS. No margin was added to the GTV to create the planning target volume (PTV). The marginal prescribed dose corresponded to the 80% isodose line. It was gradually reduced over time: 16 Gy between 1995 and 1996 (6 pts, 6.2%), 14 Gy between 1996 and 2002 (21 pts, 21.6%), 12 Gy between 2002 and 2006 (9 pts, 9.3%) and 11 Gy between 2006 and 2019 (61 pts, 62.9%). Accepted coverage limits were that 98% of PTV or more should receive at least the marginal prescribed dose. On a case-by-case basis, if nearby organs were at risk (facial and trigeminal nerves, brainstem, cochlea), the coverage prescribed dose was lowered so that 98% of PTV or more should receive at least 10 Gy. All treatment schedules were reviewed and approved by the treating radiation oncologist, neurosurgeon and physician.

### Follow-Up

Follow-up included a clinical examination and brain MRI at 6 and 12 months, then annually. An audiogram was performed annually during the first 2 years and every two years afterward. For evaluation of radiological tumor control, the last follow-up MRI images were compared to the baseline pretreatment MRI images. Tumor size was defined as the maximum mediolateral and antero-posterior diameter in transverse contrast-enhanced T1 MRI, according to the standardization of volume assessments proposed by Li et al. (15). An increase in tumor size of more than 3 mm was defined as local failure according to Huang et al. (16), two or more years after SRS (17). According to published studies, we additionally defined tumor control as freedom from re-intervention (repeated SRS or surgery) (18). All these criteria enable to take into account a known transient enlarge of tumor after SRS (19). Clinical treatment-related toxicities were defined as new neurological deficits occurring after SRS. These toxicities were classified as temporary when they resolved spontaneously or after a short course of medical therapy such as corticosteroids, and as permanent if they did not resolve. Early toxicity was defined as the appearance of a clinical sign within 90 days of the end of radiotherapy and late toxicity beyond 90 days after treatment. Toxicity of the facial nerve was defined as the appearance or worsening of facial paralysis [determined using the House-Brackmann scale (20)]. Toxicity of the trigeminal nerve was defined as the appearance or worsening of symptoms related to, in particular, hypoesthesia, paresthesia, or neuralgia in the territory of the trigeminal nerve. Hearing toxicity was defined as the worsening of hearing loss on the audiogram performed annually in patients with previously useful hearing (stages 1 and 2 of the Gardner-Robertson scale) and was not assessable in patients with pre-treatment deafness. Mean follow-up was 8.2 years (4.8 years for groups 11–12 Gy and 17.1 years for groups 14–16 Gy).

### Statistical Analysis

LC and overall survival (OS) were calculated using the Kaplan-Meir method. Time to local failure was defined as the period of time from SRS to the date of radiographic evidence of local failure at the treated site. The Cox proportional hazards model was performed to identify predictive factors of LC or toxicity. A two-sided p-value < 0.05 was considered significant. The following factors were included in the univariate analysis for LC: age, stage of tumor (Koos classification), history of surgery, presence of neurofibromatosis, GTV, marginal prescribed dose, received doses to the GTV (D_max_, D_min_ and percentage of GTV covered by the marginal prescribed isodose line) and, for toxicity, received doses to the homolateral cochlea (D_max_, D_2%_, D_moy_), to the trigeminal nerve (D_max_, D_2%_), to the brainstem (D_max_, D_2%_, V_12Gy_) and to the facial nerve (because of its proximity to the target volume and the impossibility of delineating or sparing it, the facial nerve was not contoured, the latter receiving substantially the same dose as the periphery of the target volume). The Spearman correlation enabled the identification of strongly correlated factors between them that were not included in the multivariate analysis. Factors associated with a p-value < 0.1 in the univariate analysis were included in the multivariate analysis. Finally, comparisons of LC and facial and trigeminal nerve toxicity curves were conducted using the log-rank test.

## Results

### Local Control (LC)

**Figure 1:**
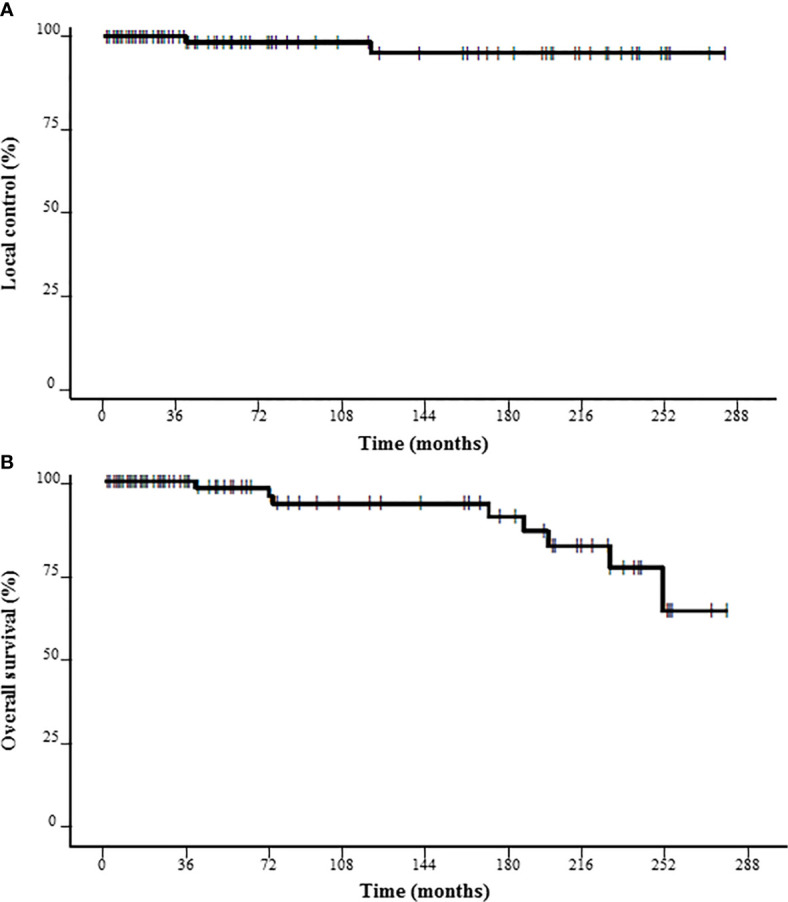
Probability of local control **(A)** and overall survival **(B)** for the 97 patients receiving SRS for vestibular schwannoma.

Following SRS, LC at 3, 5 and 10 years was 100%, 98.4%, and 95.6%, respectively (**Figure 1**). Two local failures were observed at 3 and 10 years after the end of SRS. The marginal prescribed dose was 14 Gy for these two vestibular schwannomas. Thus, LC at 3, 5 and 10 years was 100% for those with ≤ 13 Gy as the marginal prescribed dose (NS). No statistically significant predictive factor of LC was found (**Table 2**).

**Table 2:** Results of univariate and multivariate analyses for local control, overall survival and cranial nerve toxicity incidence.

	Univariate analysis									Multivariate analysis								
	Local control			Trigeminal nerve toxicity			Facial nerve toxicity			Local control			Trigeminal nerve toxicity			Facial nerve toxicity		
	HR	CI95%	p	HR	CI95%	p	HR	CI95%	p	HR	CI95%	p	HR	CI95%	p	HR	CI95%	p
**Age**	–	–	–	0.96	0.91–1.01	0.12	0.98	0.94–1.04	0.55	–	–	–	0.97	0.92–1.03	0.38	0.99	0.94–1.05	0.92
**GTV volume**	0.67	0.22–2.02	0.37	1.22	1.01–1.46	0.08	**1.25**	**1.06–1.46**	**0.02**	0.61	0.21–1.83	0.38	0.95	0.68–1.33	0.77	1.12	0.94–1.34	0.21
**Prescribed dose**	1.45	0.61–3.44	0.39	**1.7**	**1.15–2.59**	**0.01**	**1.80**	**1.21–2.69**	**0.003**	1.65	0.69–3.95	0.26	**1.31**	**0.77–2.23**	**0.049**	**1.65**	**1.05–2.59**	**0.03**
**GTV D_max_**	–	–	–	**1.13**	**1.05–1.22**	**0.01**	–	–	–	–	–	–	1.11	0.94–1.30	0.22	–	–	–
**Trigeminal nerve D_max_**	–	–	–	–	–	–	1.02	0.66–1.59	0.91	–	–	–	–	–	–	–	–	–

Bolded values are statistically significant values.

Pseudo-progression was observed in 12.4%, 3.1%, and 0% of cases at 6, 12, and 24 months, respectively. The transient increase at 6 months was significantly higher for patients with marginal prescribed doses ≥ 14 Gy (29.6% for groups 14-16 Gy vs. 7.5% for groups 11–12 Gy, p = 0.028). In the same way, perilesional edema was observed in 7.7%, 2.3%, and 0% at 6, 12, and 24 months, respectively (11.5% for groups 14–16 Gy vs. 6.2% for groups 11–12 Gy, p = 0.42).

### Overall Survival

Following SRS, OS at 3, 5 and 10 years was 100%, 98.2%, and 93.6%, respectively (**Figure 1**). OS at 3, 5, and 10 years was respectively 100%, 96.9%, and 91.8% for groups 11–12 Gy vs. 100%, 100%, and 95.7% for groups 14–16 Gy (NS). No patient died of tumor progression or SRS toxicity.

### Toxicities

Concerning toxicity to the trigeminal nerve (including hypoesthesia, paresthesia, or neuralgia in the territory of the trigeminal nerve), an onset or worsening was reported for 7 patients (7.2%): 2 pts (2.9%) and 5 pts (18.5%) for groups 11–12 Gy and 14–16 Gy, respectively (p = 0.098). These toxicities appeared in 85% of cases within the first year after SRS. No permanent toxicity was observed for groups 11 Gy, 12 Gy, and 14 Gy (vs. 33.3% for group 16 Gy). In univariate analysis, significant predictive factors of trigeminal toxicity were a higher marginal prescribed dose (HR = 1.80, 95% CI = 1.21–2.69, p = 0.003) and higher GTV (HR = 1.25, 95% CI = 1.06–1.46, p = 0.02). In multivariate analysis, a higher marginal prescribed dose remained a statistically significant predictive factor of trigeminal toxicity (HR = 1.65, 95% CI = 1.07–3.10, p = 0.028) (**Table 2**).

Concerning toxicity of the facial nerve, an onset or worsening were reported for 6 patients (6.2%): 2 pts (2.9%) and 4 pts (14.8%) for groups 11–12 Gy and 14–16 Gy, respectively (p = 0.025). Concerning subgroup analysis of groups 11–12 Gy, there was a trend toward a significant difference (1.6% vs. 11.1% for group 11 Gy vs. 12 Gy respectively, p = 0.061). As for the trigeminal nerve, toxicities appeared in 85% of cases within the first year after SRS. No permanent toxicity was observed for groups 11, 12, and 14 Gy (vs. 33% for group 16 Gy). In univariate analysis, significant predictive factors of trigeminal toxicity were a higher marginal prescribed dose (HR = 1.70, 95% CI = 1.12–2.59, p = 0.013) and a higher GTV D_max_ (HR = 1.13, 95% CI = 1.05–1.22, p = 0.007). In multivariate analysis, a higher marginal prescribed dose remained a statistically significant predictive factor of facial toxicity (HR = 1.31, 95% CI = 0.77–2.23, p = 0.049) (**Table 2**).

Concerning hearing toxicity, a hearing loss or decrease was observed in 35.7% (20 pts) of patients with pretreatment useful hearing. Five years after treatment, 13 pts (34.2%) and 7 pts (38.8%) in groups 11–12 Gy and 14–16 Gy, respectively, presented hearing loss (NS). No statistically significant predictor of hearing loss was found in univariate or multivariate analysis. Other toxicities included early side effects and consisted of headaches (1 pt, 1%) and epilepsy (1 pt, 1%).

## Discussion

To our knowledge, this study is the first to investigate the efficacy and toxicity of a de-escalation of the marginal prescribed dose of up to 11 Gy for SRS of vestibular schwannomas. It was therefore important to show the non-inferiority of a dose reduction for LC. The strength of our study also lies in the length of patient follow-up: an average of 8.2 years, including 16 years for patients with marginal prescribed doses of over 11 Gy. 5-year LC was excellent, with an absence of local failure for patients treated with a marginal prescribed dose of 11 Gy and was not significantly different from LC for patients with marginal prescribed doses of over 11 Gy (100% vs. 98%, p = 0.3).

Most publications are based on treatments performed with a CyberKnife or a GammaKnife. Only 10 studies have been published about Linac-based SRS for vestibular schwannomas (18, 21–29). The median number of patients included in these studies was 76 and outcomes were generally reported for about 5 years, with a median follow-up of 5.5 years. Moreover, there was a lack of facial toxicity data. The median marginal prescribed dose was 12.5 Gy and the 5-year LC 88.9% (range: 68%–100%, 1,204/1,356 pts). The largest published study with 335 patients and with a marginal prescribed dose of 12 Gy had reported a 5-year LC of 89% with a follow-up of 2.5 years. Concerning GammaKnife- or CyberKnife-based SRS published studies, outcomes are the same, with an LC of 95% (2691/2834 patients) within the follow-up period (12). In our series, with a mean follow up of 8.2 years, we found a 5-year LC of 98.4%. For the specific 61 patients treated with a 11 Gy marginal prescribed dose, LC was 100% with a mean follow up of 3.4 years.

The two main limitations of our study are the smaller number of patients treated with 12 to 13 Gy as the prescribed marginal dose (9 vs. 61 pts) and the shorter follow-up for those treated with 11 Gy (168 vs. 41.2 months) even if mean follow-up was 60.5 months (i.e., 5 years) for 36 patients treated with 11 Gy (range: 26.2–158 months). Therefore, it is difficult to really compare these two groups in the absence of a randomized study and the standard prescribed marginal dose in a single session should remain 12 to 13 Gy as recommended by the NCCN guidelines and RTOG studies. Other limitations of our study are that it is a retrospective study and thus dosimetric data (conformity index, gradient index, parts of received doses to organs at risk or PTV) are missing because the first SRS treatments started in 1995, i.e., over 20 years ago. However, the 20-year time span is also a strength, as it enabled a very long follow-up. The first patients treated in our institution had significantly bigger vestibular schwannomas with more pre-treatment symptoms (more stage 4, more initial trigeminal nerve damage and more hearing impairment) and a longer follow-up. Normo-FSRT can be now be used thanks to frameless masks without invasive procedures; this technique enables the treatment of easier stage 4 vestibular schwannomas as well as limiting toxicities in cases of proximity to an at-risk organ (cochlea, trigeminal nerve, or brainstem). Moreover, new and modern radiotherapy techniques such as the use of VMAT with non-coplanar arcs help to avoid organs at risk and thereby reduce toxicities. Nevertheless, it is not possible to spare the facial nerve that is attached to the tumor, whatever the technique used. Thus, it is important to try to reduce the marginal prescribed dose as much as possible.

In published studies of Linac-based SRS for vestibular schwannomas with marginal prescribed doses of 12 to 14 Gy, the trigeminal toxicity rate at 5 years was 9.7% (range: 4%–13%, 49/506 pts) of which 3.9% was permanent toxicity (range: 0%–8%, 42/1,087 pts) (18, 22–29). The three largest published studies involving more than 100 patients per study have reported a trigeminal toxicity rate of 10% at 5 years of which 4% was permanent (18, 24, 27). This trigeminal toxicity rate (transient or permanent) is about 6% (125/2,075 patients) according to GammaKnife- or CyberKnife-based SRS published studies (12). In our series, we found a trigeminal toxicity rate of 3% at 5 years of which 0% was permanent for patients with a marginal prescribed dose ≤ 12 Gy. Interestingly, only 2 transient trigeminal toxicities (3.3%) and no permanent trigeminal toxicities were reported in our study for patients treated with a marginal prescribed dose of 11 Gy.

Facial nerve toxicity was reported in 6.9% of cases in published Linac-based SRS studies (range: 2%–17%, 35/506 pts), 4.3% of which (20/465 pts) was permanent (18, 21–29). According to published studies of GammaKnife- or CyberKnife-based SRS, the facial toxicity rate was 3.6% (74/2,064 patients) (12). In our series, we found a 2.9% rate of global facial toxicity, 0% of which was permanent. Interestingly, transient facial toxicity occurred in only 1.6% of patients treated with a marginal prescribed dose of 11 Gy versus 11.1% of patients with a marginal prescribed dose of 12 Gy. Even if the follow-up was shorter for patients treated with 11 Gy as a marginal prescribed dose, we showed that 85% of toxicity occurred within the first years. Therefore, most of the toxicities likely occurred near the beginning of the follow-up period.

Finally, concerning hearing toxicity, our results are also in agreement with published studies showing about a 40% rate of hearing loss or decrease among patients with pretreatment useful hearing, whether treated with Linac-based SRS (39%, 382/989 pts) (18, 21–29) or GammaKnife- or CyberKnife-based SRS (49%, 349/716 pts) (12). It seems that this type of toxicity is not significantly influenced by the marginal prescribed dose. Perhaps, fractionation is more effective if hearing preservation is desired, but the evidence so far is scant (12). Only 2 articles reported long-term results of FSRT, with hearing deterioration in 45% of cases (62/138 patients). Trigeminal and facial toxicity is significantly higher with FSRT, with reported rates of transient and permanent toxicity at 8.4% (30/356 patients) and 11.2% (40/356 patients), respectively (30, 31).

This study, with its exceptionally long follow-up of 8.2 years, reports the excellent outcomes of Linac-based SRS for vestibular schwannomas, especially in the areas of efficacy (10-year LC of 95.6%) and safety (a 7.2% rate of transient trigeminal toxicities of which 2% were permanent, a 6.2% rate of transient facial toxicities of which 2% were permanent and a 40% rate of hearing impairment). That confirms the place of SRS in therapeutic strategies for stages 1–3 vestibular schwannomas, particularly in comparison to surgery.

## Conclusion

Linac-based SRS for stages 1–3 vestibular schwannomas provides excellent outcomes: a 10-year LC rate of over 95%, with a permanent facial or trigeminal toxicity rate of under 5%. The standard prescribed marginal dose in a single session should remain 12 to 13 Gy as recommended by the NCCN guidelines and RTOG studies. Therefore, a marginal prescribed dose of 11 Gy seems to decrease cranial nerve toxicity and facial toxicity in particular, without reducing LC. Prospective studies with longer follow-up are needed.

## Data Availability Statement

The raw data supporting the conclusions of this article will be made available by the authors, without undue reservation.

## Ethics Statement

Study ethics approval was obtained on 25 September 2020 (CECIC Rhône‐Alpes‐Auvergne, Grenoble, IRB 5921). As the study was retrospectively completed, written informed consent was waived by the ethics committee.

## Author Contributions

GD, MU, and IM analyzed the data. GD and MU wrote, revised the manuscript, and both contributed equally to this work. TK, JB, TM, PV, VC, VD, ML and J-JL also revised the manuscript. TK and JB both contributed equally to this work. All authors contributed to the article and approved the submitted version.

## Conflict of Interest

The authors declare that the research was conducted in the absence of any commercial or financial relationships that could be construed as a potential conflict of interest.

## Abbreviations

CT, Computed Tomography; GTV, Gross Tumor Volume; LC, Local Control; Linac, Linear Accelerator; MRI, Magnetic Resonance Imagery; NF2, Neurofibromatosis type 2; OS, Overall Survival; PTV, Planning Target Volume; SRS, Stereotactic Radiosurgery.
